# Validation of the MISSCARE-BRASIL survey - A tool to assess missed nursing care ^1^

**DOI:** 10.1590/1518-8345.2354.2975

**Published:** 2017-12-21

**Authors:** Lillian Dias Castilho Siqueira, Maria Helena Larcher Caliri, Vanderlei José Haas, Beatrice Kalisch, Rosana Aparecida Spadoti Dantas

**Affiliations:** 2 PhD, RN, Hospital Universitário, Universidade Federal da Grande Dourados, Dourados, MS, Brazil.; 3 PhD, Associate Professor, Escola de Enfermagem de Ribeirão Preto, Universidade de São Paulo, PAHO/WHO Collaborating Centre for Nursing Research Development, Ribeirão Preto, SP, Brazil.; 4 PhD, Visitor Professor, Universidade Federal do Triângulo Mineiro, Uberaba, MG, Brazil.; 5 PhD, Full Professor, School of Nursing, University of Michigan, Ann Arbor, Michigan, United States of America.; 6 PhD, Full Professor, Escola de Enfermagem de Ribeirão Preto, Universidade de São Paulo, PAHO/WHO Collaborating Centre for Nursing Research Development, Ribeirão Preto, SP, Brazil.

**Keywords:** Validation Studies, Nursing Care, Nursing Methodology Research, Patient Safety, Nursing, Factor Analysis, Statistical

## Abstract

**Objective:**

to analyze the metric validity and reliability properties of the MISSCARE-BRASIL survey.

**Method:**

methodological research conducted by assessing construct validity and reliability via confirmatory factor analysis, known-groups validation, convergent construct validation, analysis of internal consistency and test-retest reliability. The sample consisted of 330 nursing professionals, of whom 86 participated in the retest phase.

**Results:**

of the 330 participants, 39.7% were aides, 33% technicians, 20.9% nurses, and 6.4% nurses with administrative roles. Confirmatory factorial analysis demonstrated that the Brazilian Portuguese version of the instrument is adequately adjusted to the dimensional structure the scale authors originally proposed. The correlation between “satisfaction with position/role” and “satisfaction with teamwork” and the survey’s missed care variables was moderate (Spearman’s coefficient =0.35; p<0.001). The results of the Student’s t-test indicated known-group validity. Professionals from closed units reported lower levels of missed care in comparison with the other units. The reliability showed a strong correlation, with the exception of “institutional management/leadership style” (intraclass correlation coefficient (ICC)=0.15; p=0.04). The internal consistency was adequate (Cronbach’s alpha was greater than 0.70).

**Conclusion:**

the MISSCARE-BRASIL was valid and reliable in the group studied. The application of the MISSCARE-BRASIL can contribute to identifying solutions for missed nursing care.

## Introduction

The hospital work environment has been described as fast-paced and unpredictable, favoring interruptions and errors in nursing care, as these professionals are constantly moving from one activity to the next and managing information from several sources. Furthermore, they tend to multitask with high rates of interruptions^1^. Faced with multiple demands and insufficient resources, professionals can find it impossible to meet all nursing care requirements, and by prioritizing, they may choose to leave some aspects of care unfinished in many situations. In such circumstances, they may abbreviate, delay or simply omit the care^2^.

The phenomenon of omitted or missed care in nursing is defined as any aspect of required patient care that is delayed or omitted (either in part or entirely)^2^. This concept was first described in a qualitative study^3^, which conducted 25 focus groups with medical-surgical nursing staff in medical-surgical units (107 nurses, 15 nursing technicians, and 51 nursing aides) at two hospitals in the United States. The participants were separated by professional category and were asked about missed care in their work environment and reasons for such omissions. The author identified nine themes relative to a lack of nursing care as follows: ambulation, turning, delayed or missed feedings, patient teaching, discharge planning, emotional support, hygiene, intake and output documentation, and surveillance. Seven themes relative to the reasons for missing care were reported as follows: staff issues, amount of time for complete nursing interventions, poor use of existing staff resources, “It’s not my job” syndrome, ineffective delegation, habit, and denial^3^.

Authors who have analyzed the concept of missed nursing care suggest that this phenomenon can negatively influence patient outcomes, threatening patient safety^2^. They have shown that failure to turn, bathe, and ambulate bedridden patients may contribute to pressure injuries and patient weakness, and that missed mouth care with critically ill patients can increase the risk of pneumonia^2^. Thus, using an instrument capable of investigating the extent and nature of this phenomenon is essential, making it possible to study staff perceptions of care omission and reasons for such omission. 

To this end, the MISSCARE survey has been developed to measure missed nursing care and to analyze its causes. The tool was developed and validated in the United States with a sample of 1,098 nursing professionals and consists of items that comprise 24 basic nursing care elements in part A and 17 reasons for missed care in part B^4^. 

In Brazil, the MISSCARE survey has already been culturally adapted and tested for internal consistency^5^. A different study expanded the original instrument to include other items associated with missed nursing care and reasons for missed care specific to the Brazilian context (unpublished data). This instrument is called MISSCARE-BRASIL.

The aim of this study was to validate the MISSCARE-BRASIL survey to enable investigations about the phenomenon in the Brazilian context. 

## Method

The study was methodological and cross-sectional. The MISSCARE-BRASIL survey was validated by assessing psychometric properties related to its construct validity and reliability via confirmatory factor analysis, known-groups validation, convergent construct validation, analysis of internal consistency (Cronbach’s alpha) and test-retest reliability. 

This study abided by ethical precepts, as per Resolution 466/2012 of the Brazilian National Health Council and was approved by the local research ethics committee (protocol CAAE no.38506614000005393). The primary author granted permission for the survey to be validated and used in Brazil. 

The original MISSCARE survey includes 41 items that quantify missed nursing care. The survey includes a cover page with questions about participants’ demographic and professional characteristics; part A, with 24 items related to omitted or missed care; and part B, with 17 items on reasons for missed nursing care. Part B contains three factors or domains as follows: labor resources (five items), material resources (three items), and communication/teamwork (nine items) The MISSCARE-BRASIL survey includes 28 items in part A and 28 items in part B.

The items in part A are answered on a Likert-type scale and scored from one to five, with one representing the highest level of missed care and five representing no missed care. In part B, the items are scored from one to four, with one corresponding to “a significant factor” and four to “not a reason for unmet nursing care”.

The study was conducted at a large-scale teaching hospital in the state of São Paulo, Brazil. The hospital is a state autarchy associated with the University of São Paulo that is used for provision of teaching, research and medical hospital care. A simple random sample was drawn from the population frame. The participants were selected according to the following inclusion criteria: nurses, nursing technicians, and nursing aides with an employment relationship with the selected hospital, assigned to its various units, and who had worked in the sector for at least a month. Professionals on vacation or medical leave were excluded.

Sample size was determined by considering an adequate sample balance and consensus to estimate the minimum sample necessary to conduct a reliable confirmatory factor analysis^6^. With prior knowledge of the target population, which consisted of 1618 nursing professionals, a minimum sample of 300 nursing professionals (nurses, technicians and aides) was initially established. A value of 20% sampling loss was included to account refusal to participate, vacation, medical leave, or other leaves from work. To analyze test-retest reliability, intraclass correlation coefficient (ICC) was set at ICC=0.7 among omission scores, allowing a value equal to or greater than 0.5 for a statistical power of 90%, with α=0.05 significance level. Using Power Analysis and Sample Size (PASS) version 13, a minimum number of 86 participants was established. 

MISSCARE-BRASIL is a self-completion survey. After complying with the required ethical procedures, the questionnaire was given directly to participants in a brown envelope. The surveys were returned at a later time, and anonymity was ensured. However, a code was assigned to each participant, so that those selected to participate in the retest phase could be located. Retesting was conducted with 86 professionals two weeks after the first test. According to the researcher’s estimates, this was enough time for participants to forget the answers they gave the first time around.

After applying MISSCARE-BRASIL in the selected sample, statistical tests were conducted using Statistical Package for Social Sciences (SPSS) software, version 17.0. Imputations were carried out in parts A and B to fill in missing values and to increase the total number of cases, substituting the mean of the other items for nonresponses. The answers to items from both part A and B were reverse-scored, with (1=5) (2=4) (3=3) (4=2) (5=1) for part A, and (1=4) (2=3) (3=2) (4=1) for part B, as instructed by the author of the original survey via e-mail. 

The aim was to verify whether the Brazilian version of the survey measured the studied phenomenon clearly and reliably, reaching the desired objectives. To this end, the answers were submitted to confirmatory factor analysis (CFA) through the application or add-on module of the International Business Machines (IBM) SPSS and Analysis of Moment Structures (AMOS), version 16. CFA was conducted using structural equation models, with the 28 items in part B of the MISSCARE-BRASIL to confirm the factor structure of missed care ratios. The original survey had the following three factors: material resources, labor resources, and communication. The MISSCARE-BRASIL survey includes these three factors and two more as follows: ethical dimensions and styles of institutional management and leadership. In the present study, the model was adjusted considering criteria that sought to determine similarities among the observed variance-covariance matrixes in the sample and that were predicted by the model being tested. Successfully obtaining an adequate model for observation confirms instrument validity.

To verify convergent construct validity, positive correlations were hypothesized between the level of professional satisfaction and the MISSCARE-BRASIL missed care variables via Spearman’s coefficient. Coefficients <0.30 represent weak correlation, between 0.30 and 0.49, moderate, and equal to or greater than 0.50, strong. 

To test construct validity through known groups, mean scores for missed nursing care (per participant) were calculated and analyzed. This total score was the mean missed care score identified for each of the nursing care elements presented in part A. The hypothesis was that nursing professionals who worked in closed sectors presented higher levels of job satisfaction, were not planning to leave their positions, and would present fewer missed nursing care elements. Other items related to missed care were treated as dichotomous variables. Care elements were considered missed if the answers were “occasionally,” “rarely” or “never.” The Student’s t-test for independent samples was used to compare the existence or lack of differences among group means or the study criteria. Furthermore, Cohen’s d was used to classify the distance between means as small (d<0.20), medium (≥0.20 to <0.50) or large (≥0.50).

Cronbach’s alpha was used to estimate internal consistency, with satisfactory values set at >0.70^7^. To analyze the instrument’s stability, the ICC was used; values below 4 indicated low reliability, between 0.4 and 0.74, moderate to good, and equal to or greater than 0.75 indicated excellent reliability. All inferential analyses were conducted based on a 5% significance level (α=0.05).

## Results

Three hundred thirty nursing professionals participated in the study; 131 (39.7%) were aides, 109 (33%) technicians, 69 (20.9%) nurses, and 21 (6.4%) nurses with administrative roles. The mean age was 39.9 years, and 255 participants (77.3%) were women. In terms of the highest educational degree obtained, except for the 90 nurses, among the other participants, most had finished secondary education (183; 55.5%), and nursing technician school (140; 42.4%). Most (95.5%) of the professionals worked more than 30 hours/week, and had over ten years at the job (52.1%) and over five years of experience in the inpatient sector (54.8%). Over 80% of the staff did not have plans to leave their positions or current roles in the following year. 

In terms of construct validity analysis, this analysis was conducted with AMOS software, showing regression coefficients and factor loading for all five factors. Factor loadings for “communication” were between 0.54 and 0.71. “Material resources” ranged between 0.60 and 0.78, and “labor resources”, 0.46 to 0.71. The factor “ethical dimensions” presented factor loadings between 0.78 and 0.81 and, finally, factor loadings for “management” fell between 0.55 and 0.78. The comparative fit index (CFI) achieved in this work was CFI ~ 0,9. Table 1 shows the results of CFA, i.e., factor analysis for the MISSCARE-BRASIL survey.

**Table 1:** Confirmatory Factor Analysis of MISSCARE-BRASIL*,* Ribeirão Preto, SP, Brazil, 2015

			**Unstandardized regression coefficients**	**Standard-error**	**Critical ratio**	**Factor** **loading**	p*
**Item B11**	**←**	**Communication**	**1**				**0,670**
**Item B5**	**←**	**Communication**	**0,865**	**0,091**	**9,473**	**0,574**	**<0,001**
**Item B16**	**←**	**Communication**	**1,034**	**0,097**	**10,708**	**0,658**	**<0,001**
**Item B15**	**←**	**Communication**	**1,08**	**0,099**	**10,896**	**0,671**	**<0,001**
**Item B13**	**←**	**Communication**	**1,06**	**0,076**	**13,94**	**0,712**	**<0,001**
**Item B8**	**←**	**Communication**	**0,833**	**0,093**	**8,958**	**0,541**	**<0,001**
**Item B14**	**←**	**Communication**	**0,994**	**0,092**	**10,794**	**0,665**	**<0,001**
**Item B7**	**←**	**Communication**	**0,858**	**0,093**	**9,204**	**0,557**	**<0,001**
**Item B12**	**←**	**Communication**	**0,985**	**0,09**	**10,996**	**0,678**	**<0,001**
**Item B24**	**←**	**Communication**	**0,976**	**0,095**	**10,289**	**0,629**	**<0,001**
**Item B10**	**←**	**Material resources**	**1**				**0,733**
**Item B6**	**←**	**Material resources**	**0,881**	**0,082**	**10,773**	**0,645**	**<0,001**
**Item B9**	**←**	**Material resources**	**1,092**	**0,069**	**15,854**	**0,783**	**<0,001**
**Item B23**	**←**	**Material resources**	**0,945**	**0,094**	**10,047**	**0,605**	**<0,001**
**Item B1**	**←**	**Labor resources**	**1**				**0,611**
**Item B4**	**←**	**Labor resources**	**1,076**	**0,081**	**13,23**	**0,663**	**<0,001**
**Item B3**	**←**	**Labor resources**	**0,816**	**0,101**	**8,087**	**0,529**	**<0,001**
**Item B2**	**←**	**Labor resources**	**0,736**	**0,101**	**7,282**	**0,467**	**<0,001**
**Item B17**	**←**	**Labor resources**	**1,139**	**0,112**	**10,155**	**0,713**	**<0,001**
**Item B19**	**←**	**Labor resources**	**0,966**	**0,11**	**8,755**	**0,584**	**<0,001**
**Item B27**	**←**	**Labor resources**	**1,091**	**0,117**	**9,359**	**0,638**	**<0,001**
**Item B28**	**←**	**Labor resources**	**1,051**	**0,118**	**8,937**	**0,603**	**<0,001**
**Item B18**	**←**	**Ethical dimension**	**1**				**0,817**
**Item B20**	**←**	**Ethical dimension**	**0,971**	**0,065**	**15,043**	**0,781**	**<0,001**
**Item B25**	**←**	**Ethical dimension**	**1,047**	**0,066**	**15,829**	**0,818**	**<0,001**
**Item B21**	**←**	**Management**	**1**				**0,763**
**Item B22**	**←**	**Management**	**0,986**	**0,075**	**13,158**	**0,78**	**<0,001**
**Item B26**	**←**	**Management**	**0,787**	**0,086**	**9,118**	**0,553**	**<0,001**

*p - probability, p-value

Convergent validation was verified by positive correlations between levels of professional satisfaction and missed care variables of the MISSCARE-BRASIL instrument. The correlation between “satisfaction with position/role” and “satisfaction with teamwork” and the survey’s missed care variables was moderate (Spearman’s coefficient=0.35; p<0.001). There was a weak correlation between the variable “satisfaction with profession” and missed care variables, with Spearman’s correlation coefficient ranging between 0.22 and 0.24. These positive correlations indicate that the variables covaried in the same direction, i.e., the greater the dissatisfaction, the greater the number of missed care elements (positive correlation). 

The results of the Student’s t-test indicated known-group validity. Professionals with the highest levels of satisfaction and those who did not plan to leave their positions/roles perceived fewer missed nursing care elements (lower means) in their units and, similarly, as hypothesized, professionals from closed units reported lower levels of missed care in comparison with the other units. These results are presented in Table 2.

**Table 2:** Results of known-group validity of the MISSCARE-BRASIL survey, considering professional satisfaction with position, profession and teamwork, intention to leave position/role and comparison between sectors. Ribeirão Preto, SP, Brazil, 2015

**Variables**			**Mean missed care score**				**Number of missed care elements per participant**			
		**n (%)**	**Mean**	SD*	**p** ^**†**^	**d** ^**‡**^	**Mean**	SD*	**p** ^**†**^	**d** ^**‡**^
**Satisfaction with position/role**										
	**Satisfied**	**205 (62,1)**	**1,70**	**0,40**	**<0,001**	**0,79**	**3,6**	**3,47**	**<0,001**	**0,82**
	**Dissatisfied**	**125 (37,8)**	**2,05**	**0,51**			**7,0**	**5,32**		
**Satisfaction with profession**										
	**Satisfied**	**250 (75,8)**	**1,77**	**0,44**	**<0,001**	**0,57**	**4,34**	**4,22**	**0,001**	**0,50**
	**Dissatisfied**	**80 (24,2)**	**2,03**	**0,52**			**6,57**	**5,21**		
**Satisfaction with teamwork**										
	**Satisfied**	**179 (54,2)**	**1,72**	**0,45**	**<0,001**	**0,54**	**3,86**	**4,10**	**<0,001**	**0,50**
	**Dissatisfied**	**149 (45,2)**	**1,97**	**0,47**			**6,08**	**4,83**		
**Plans to leave position/role**										
	**Yes**	**60 (18,1)**	**2,1**	**0,60**	**0,007**	**0,67**	**7,12**	**6,26**	**<0,001**	**0,65**
	**No**	**267 (80,9)**	**1,79**	**0,43**			**4,31**	**3,88**		
**Sector**										
	**Closed units** ^**§**^	**68 (20,6)**	**1,66**	**0,44**	**0,001**	**0,47**	**3,44**	**3,52**	**0,001**	**0,41**
	**Others**	**262 (79,4)**	**1,88**	**0,47**			**5,26**	**4,74**		

*SD - standard deviation; ^†^p - probability, p-value; ^‡^d - Cohen’s d (effect size); ^§^Closed units - intensive care unit/coronary care unit, immunotherapy unit, bone marrow transplant unit, oncology, hematology/chemotherapy

Test-retest reliability of the MISSCARE-BRASIL survey was measured via factor stability and presented strong positive correlations (communication ICC=0.62; p<0.001; material resources ICC=0.53; p<0.001; labor resources ICC=0.66; p<0.001; ethical dimension ICC=0.64; p<0.001), with the exception of “institutional management/leadership style” (ICC=0.15; p=0.04). Reliability was also tested by assessing the internal consistency of part A and the five factors in part B. Cronbach’s alpha was greater than 0.70, an acceptable level of internal consistency. 

## Discussion

The aim of this methodological study was to perform a psychometric evaluation of the adapted and expanded version of the MISSCARE survey, MISSCARE-BRASIL, for use with Brazilian nursing professionals. The motivation to conduct the present study was based on evidence in the Brazilian literature of the absence of valid and reliable instruments to measure the phenomenon of missed nursing care. The international literature has shown that the results of assessments with this tool can be used to underpin nursing service management since nursing actions contribute significantly to quality of health care and, consequently, patient safety.

As in the original United States studies^4^ ^,^ ^8^, the results of this Brazilian study showed a predominance of female professionals with over 10 years of experience and a 30-hour workweek. In the United States, most professionals held a baccalaureate degree, while in Brazil, most were nursing technicians, a finding that demonstrates the differences in educational background of nursing professionals in the two countries.

The confirmatory factor analysis yielded a 5-factor model, two more than the original three factors; the new items yielded two additional factors as follows: ethical dimension and institutional management/leadership style. The resulting factor loadings established which items belonged to each factor. It is worth noting that when the authors developed the original instrument using exploratory factor analysis, it yielded three factors in part B as follows: communication/teamwork, labor resources and material resources^4^. 

In terms of known-groups validity, regarding the results of the validation in Brazil and the development of the original instrument in English, both studies hypothesized that nursing professionals who worked in closed units (such as intensive care units) would report fewer missed nursing care elements. This hypothesis is justified by the lower nurse/patient ratio in intensive care units: “1:1” or “1:2,” while rehabilitation unit nurses tend to care for more patients^4^. As predicted, in both studies, the answers given in the United States by closed unit professionals were different from those working in rehabilitation units, and in Brazil, they were different from those of professionals in open units. There were fewer reports of care omission in closed units, therefore displaying known-groups validation in both countries.

In the present study, considering convergent validation, the analysis of Spearman’s correlation coefficient showed moderate correlations between “satisfaction with position/role” and “satisfaction with teamwork” and several MISSCARE-BRASIL omission variables. Correlation between “satisfaction with profession” and omission variables was weak, with Spearman’s correlation coefficient ranging from 0.22 to 0.24. 

Internal consistency, estimated by Cronbach’s alpha, was acceptable in both the Brazilian and American studies^4^ ^,^ ^8^, as was test-retest reliability, which indicated reliable and stable measures. All five factors of the MISSCARE-BRASIL had alpha coefficients ranging between 0.77 and 0.90, indicating internal consistency. 

Considering only the three original factors of part B, alpha values were similar to the values obtained in the original study, since the “communication” factor presented the highest value^4^ ^,^ ^8^. Furthermore, although the alpha coefficient for part A was calculated, the author of the original instrument stated it was not appropriate to assess it psychometrically using the coefficient or CFA, as this part contains a list of independent nursing actions. 

Test-retest reliability showed evidence of temporally stable measures with adequate ICC values, similar to those of the original survey, which were 0.87 for part A and 0.86 for part B^4^ ^,^ ^8^. It is worth highlighting that the ICC for “institutional management/leadership style” was low, which can be explained by the means obtained in the test and retest steps that differed considerably. 

Missed nursing action is an important concept that, in part, can explain negative outcomes for hospitalized patients, such as pressure injuries. This concept is particularly pertinent and can underpin the implementation of managerial measures that strengthen human resources within organizations with the specific staff size and competencies needed to provide ongoing, safe, patient-centered care and to avoid missed care and its impact on care outcomes^9^. 

A qualitative study conducted in Brazil with nursing professionals investigated their stance before care responsibilities and found that they did not have clear criteria about the activities they had to carry out and the decisions they had to make in relation to patient needs. On average, these professionals took 4 to 30 minutes to respond to patient or family call lights, a period that may seem insignificant to busy workers but represents an “eternity” to patients. In times of scarce staffing, there is an understanding among professionals that priority should be given to drug administration and vital sign monitoring to the detriment of other activities. Additionally, the participants mentioned dissatisfaction with their own practice and lack of commitment of some nursing team members. They also indicated an unsuitable choice of profession as a possible personal factor and one of the causes of lack of commitment and identification with the profession^10^.

In this context, the measure provided by the MISSCARE-BRASIL survey has potential applications to clinical practice and research. It can be used to identify specific situations related to missed nursing care that pose challenges to quality nursing care. With this information, the actions of healthcare professionals can be redirected to reach solutions. 

The results of the present study and others that focus on missed care show that it is a global phenomenon, and it may call attention to the importance of a system-centered explanatory model for error, in which flaws are the combined result of active errors (omissions, distractions, noncompliance with norms, mistakes and forgetting) and latent conditions, such as work overload, poor task definition, insufficient supervision, communication flaws, obsolete resources, unsuitable maintenance of facilities, reduced process standardization, deficient professional training, pressures of healthcare work, and deficient technology^11^. In light of these findings, the instrument can be used in studies that aim to produce more in-depth knowledge about the mediating and/or moderating variables of this complex phenomenon. 

One limitation of the present study was that data was collected from only one public teaching hospital in the state of São Paulo. Multicenter studies with larger samples should be conducted, including professionals from both public and private hospitals in other cities and even other regions of Brazil, to take into account differences in professional nursing practice. Another limitation was the use of a self-reported method of data collection. Some answers may not have been completely accurate, since the survey investigates negative aspects of care. This means that some participants may not have been willing to indicate missed care, despite the careful attention given to anonymity. However, the high cost of directly observing a large number of nurses limited the present study’s methodological design and ability to expand data collection to include other sources of information. Further studies should be conducted using a multimethod approach. 

## Conclusions

The results of the present study showed moderate evidence of the validity and reliability of the MISSCARE-BRASIL survey when adapted to nursing professionals at a public teaching hospital in the municipality of Ribeirão Preto, São Paulo, Brazil. 

In this context, making this instrument available in Brazil aims to fill the need for tools that assess missed nursing care. However, Brazil is rich in cultural diversity, so more studies should be conducted to test the adapted version of the survey with different population samples. This means that assessing its use in clinical practice depends on further research in different hospital contexts in Brazil, including private hospitals.

The application of the MISSCARE-BRASIL survey can contribute to not only research on missed nursing care in Brazilian health institutions but also identifying solutions for this phenomenon together with the professionals involved in such care.
